# Supplementary material for: Application of Nicotinamide to Culture Medium Improves the Efficiency of Genome Editing in Hexaploid Wheat
Source: Int J Mol Sci. 2023 Feb 23;24(5):4416. doi: 10.3390/ijms24054416 (PMC10002385; doi:10.3390/ijms24054416)
Supplement: Supplementary file 1 [file ijms-24-04416-s001.zip › ijms-2203368-supplementary.pdf]

Supplementary information

Table S1. Information of SgRNA and primers used in this study.

The target gene	The sequences of SgRNA	The sequences of primers
bar		BarF: ACCATCGTCAACCACTACATCG<br>BarR: GCTGCCAGAAACCACGTCATG
GUS	ATGTCCTCGACGTACGT AAACGG	GUS406F: CAAGGAAATCCGCAACCATATC<br>GUS1401R: TCAAACGTCCGAATCTTCTCCC
Waxy-4A	AAGACCAAGGAGAAGA TCTATGG	4AWaxyF: CCCCGAAGCAACAAAGCCGGAAAG<br>4AWaxyR: TGCAGAACGCTACCTGGACATG
Waxy-7A		7AWaxyF: GAAACCGCACCGATTCGACCGGCG<br>7AWaxyR: AGTTGTTCTTGATCTTACCGTAGG
Waxy-7D		7DWaxyF: GTGCCTCTCCATGGTGGTGCGCGC<br>7DWaxyR: TGAACCGCAAAATTGATATGCCTG
Waxy-ABE-7A	AAGACCAAGGAGAAGA TCTATGG	7AWaxyF: GAAACCGCACCGATTCGACCGGCG<br>7AWaxyR: AGTTGTTCTTGATCTTACCGTAGG
Waxy-ABE-7D		7DWaxyF: GTGCCTCTCCATGGTGGTGCGCGC<br>7DWaxyR: TGAACCGCAAAATTGATATGCCTG

Supplementary Figures

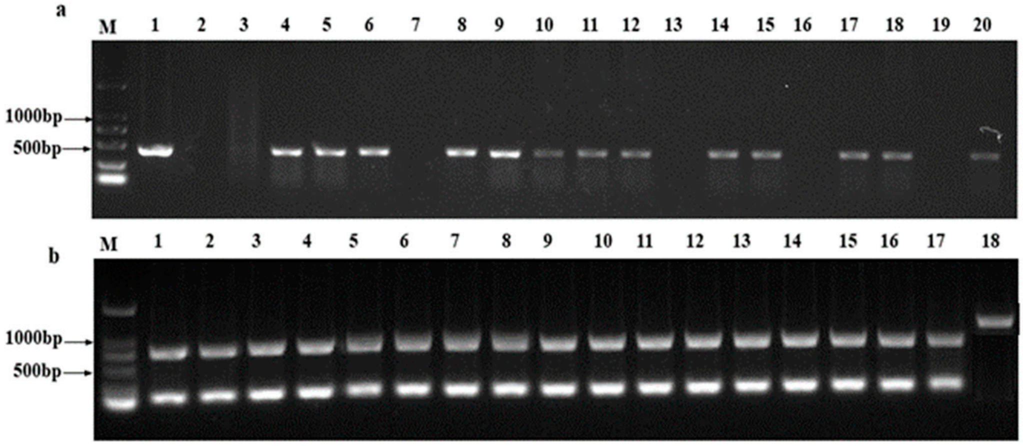

Figure S1. Detection of *bar* gene and mutant types of *GUS* gene in T_1 transgenic plants.

(a) PCR identification of *bar* gene in T_1 putatively transgenic plants. M: 2kb DNA marker; 1: positive control (plasmid); 2: negative control (water); 3-20: T_1 putatively transgenic plants. (b) PCR-RE detection of *GUS* gene in T_1 generation transgenic plants. M: 2 kb DNA marker; 1-16: T_1 generation putatively transgenic plants; 17: digested PCR products (H29); 18: undigested PCR products (H29)

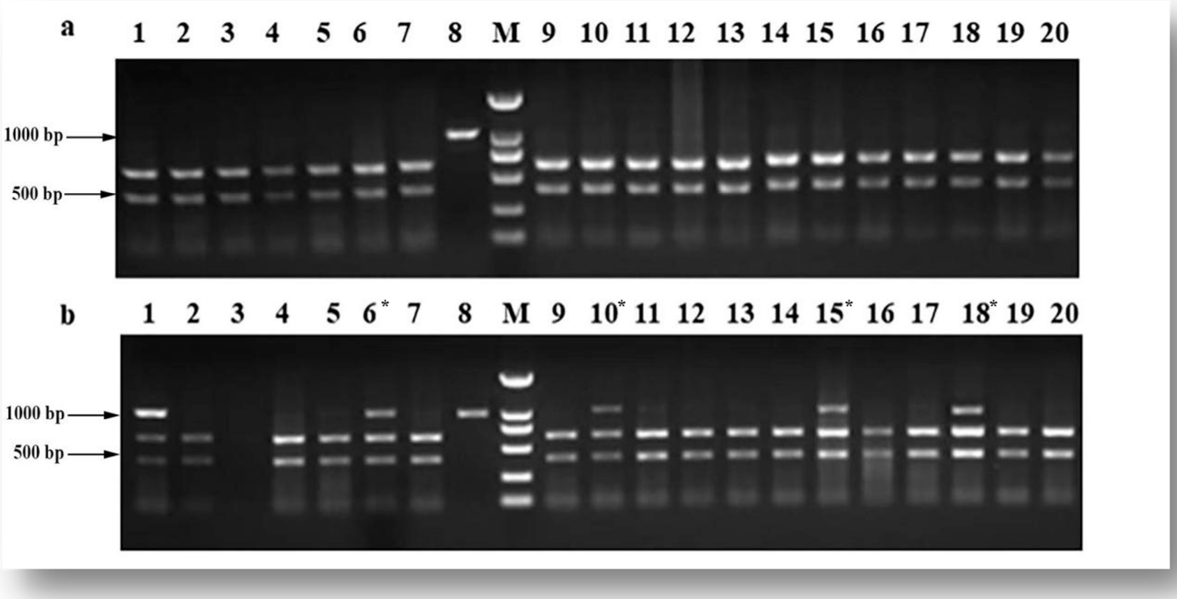

Figure S2. Detection of *GUS* gene mutations in T_2 generations treated with nicotinamide on immature embryos by PCR-RE.

(a) Control group (0 mM nicotinamide); M: 2 kb DNA marker; 1-7: 2-CK-G1; 8: undigested PCR product; 9: SnaBI digested PCR products of GUS gene transgenic lines H29; 10-15: 7-CK-G1; 16-20: 14-CK-G1. (b) Treatment groups (2.5 and 5 mM nicotinamide); M: 2 kb DNA markers; 1-4: 7-2.5-G1; 5-7: 7-5-G1; 8: undigested PCR product; 9: SnaBI digested PCR products of *GUS* gene transgenic plants in H29; 10-12: 7-5-G1; 13-16: 14-2.5-G1; 17-20: 14-5-G1; b1, b6, b10, b15 and b18: heterozygous mutants; the other transgenic plants: non-mutant plants. The mutants were marked with*.

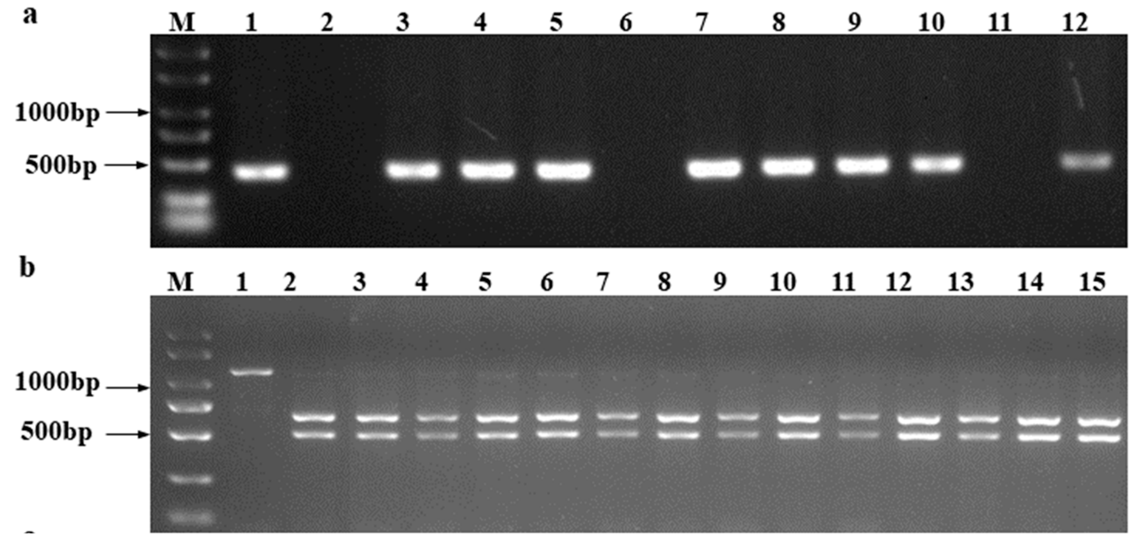

Figure S3 Detection of *bar* gene and mutation type of *TaWaxy* gene in T_0 transgenic plants.

(a): PCR detection of *bar* gene in T_0 putatively transgenic plants. M: 2 kb DNA marker; 1: positive control (plasmid); 2: negative control (water); 3-12: T_0 putatively transgenic plants. (b): PCR-RE detection of unedited material of *TaWaxy g*ene in T_0 transgenic plants. M: 2 kb DNA marker; 1: undigested PCR products (Ningchun4); 2: digested PCR products (Ningchun4) ; 3-15: T_0 transgenic plants.

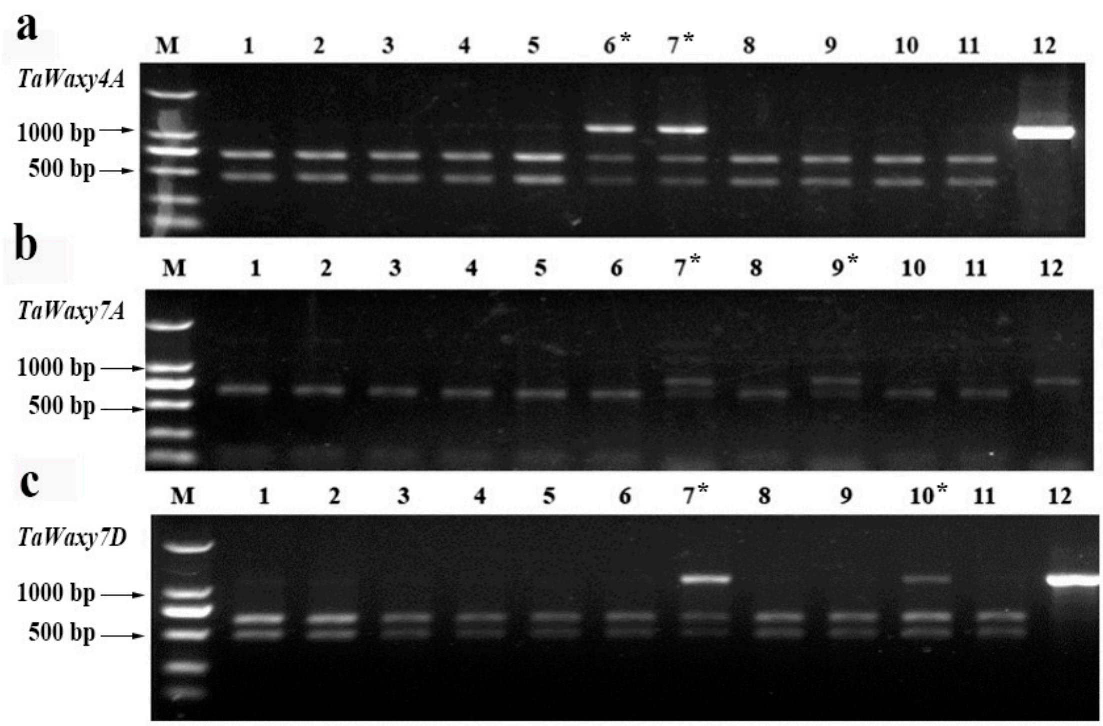

Figure S4. Mutation detection of *TaWaxy* genes in T_1 generations after nicotinamide treatment on immature embryos.

(a), (b), and (c): PCR-RE detection of the *TaWaxy* genes on chromosomes 4A, 7A, and 7D, respectively. M: 2 kb DNA markers; 1-5: 14-CK-W1; 6-8: 14-2.5-W1; 9-10: 14-5-W1; 11: *BglII* digested PCR products (Ninghcun4); 12: undigested PCR products (Ningchun4); a6, a7, b7, b9, c7 and c10: heterozygous mutants; the other transgenic plants: non-mutant plants. The mutants were marked with*.

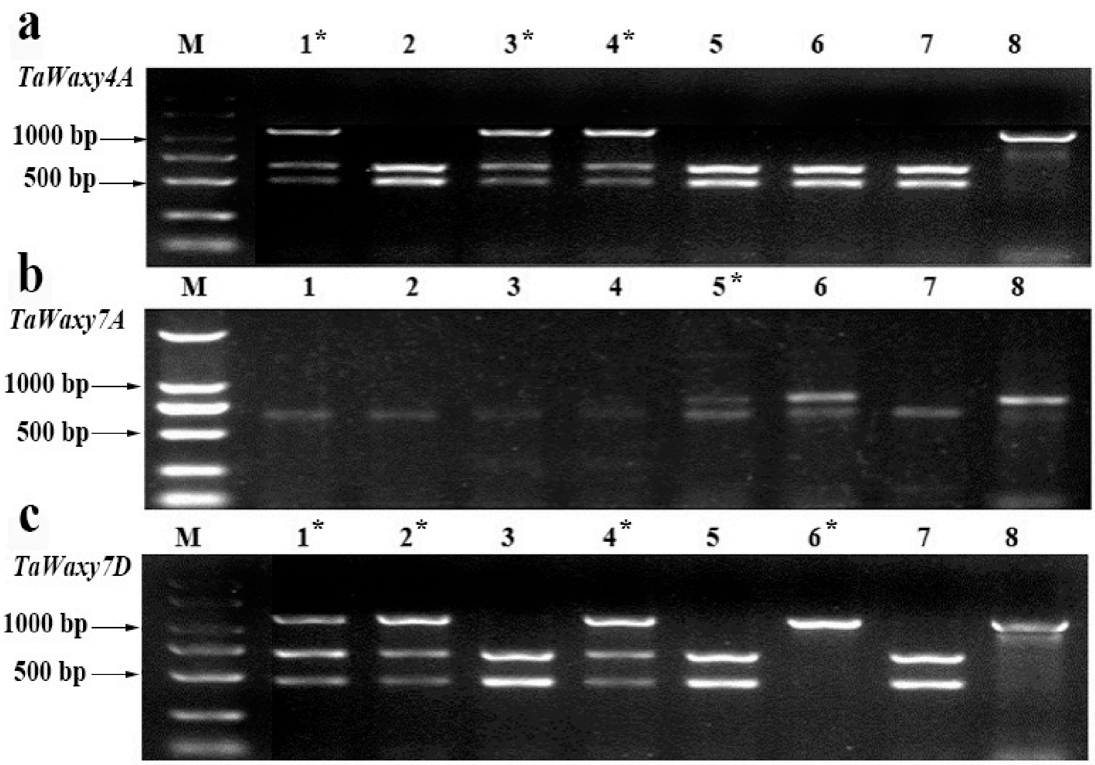

Figure S5. Detection of the mutation types for *TaWaxy* genes in T_1 plants which only had a single edited locus after nicotinamide treatment on immature embryos.

(a), (b), and (c): PCR-RE detection of *TaWaxy* genes on 4A, 7A, and 7D in order in T_1 generation. M: 2 kb DNA marker; 1-2: XD359-23 (edited locus on 7D); 3-4: XD350-8 (edited locus on 4A); 5-6: XD350-13 (edited locus on 7D); 7: *BglII* digested PCR products (Ningchun4); 8: undigested PCR products (Ningchun4); a1, a2, a3, b5, b6, c1, c2 and c4: heterozygous mutants; c6: biallelic mutants; the other transgenic plants: nonmutant plants. The mutants were marked with*.

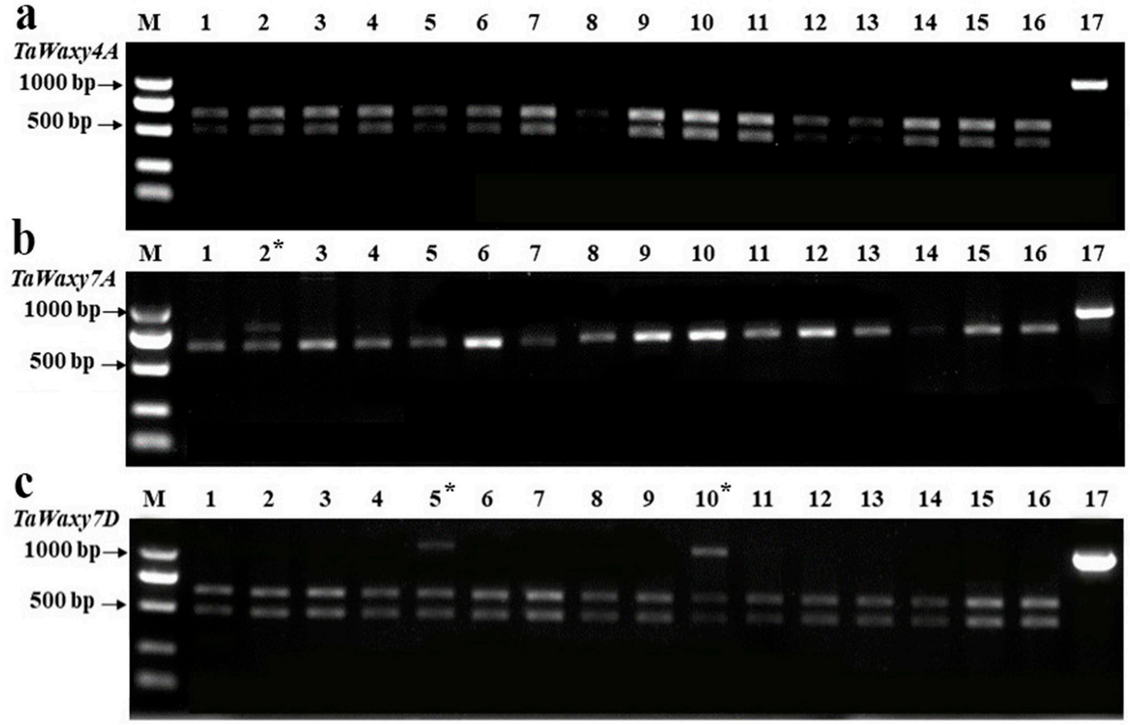

Figure S6. Detection of mutation types in T_2 plants after nicotinamide treatment on mature embryos.

(a), (b), and (c): PCR-RE detection of *TaWaxy* genes on chromosomes 4A, 7A, and 7D in T_2 generation, respectively. M: 2 kb DNA markers; 1-5: 14-2.5-W3; 6-10: 14-5-W3; 11-15: 14-CK-W3; 16: *BglII* digested PCR products (Ningchun4); 17: undigested PCR products (Ningchun4); b2, c5 and c10: heterozygous mutants; the other transgenic plants: non-mutant plants. The mutants were marked with*.
